# Arp2/3 and VASP Are Essential for Fear Memory Formation in Lateral Amygdala

**DOI:** 10.1523/ENEURO.0302-16.2016

**Published:** 2016-11-29

**Authors:** Sreetama Basu, Irina Kustanovich, Raphael Lamprecht

**Affiliations:** Sagol Department of Neurobiology, Faculty of Natural Sciences, University of Haifa, Haifa 3498838, Israel

**Keywords:** amygdala, Arp2/3, fear conditioning, formins, learning and memory, VASP

## Abstract

The actin cytoskeleton is involved in key neuronal functions such as synaptic transmission and morphogenesis. However, the roles and regulation of actin cytoskeleton in memory formation remain to be clarified. In this study, we unveil the mechanism whereby actin cytoskeleton is regulated to form memory by exploring the roles of the major actin-regulatory proteins Arp2/3, VASP, and formins in long-term memory formation. Inhibition of Arp2/3, involved in actin filament branching and neuronal morphogenesis, in lateral amygdala (LA) with the specific inhibitor CK-666 during fear conditioning impaired long-term, but not short-term, fear memory. The inactive isomer CK-689 had no effect on memory formation. We observed that Arp2/3 is colocalized with the actin-regulatory protein profilin in LA neurons of fear-conditioned rats. VASP binding to profilin is needed for profilin-mediated stabilization of actin cytoskeleton and dendritic spine morphology. Microinjection of poly-proline peptide [G(GP_5_)_3_] into LA, to interfere with VASP binding to profilin, impaired long-term but not short-term fear memory formation. Control peptide [G(GA_5_)_3_] had no effect. Inhibiting formins, which regulate linear actin elongation, in LA during fear conditioning by microinjecting the formin-specific inhibitor SMIFH2 into LA had no effect on long-term fear memory formation. We conclude that Arp2/3 and VASP, through the profilin binding site, are essential for the formation of long-term fear memory in LA and propose a model whereby these proteins subserve cellular events, leading to memory consolidation.

## Significance Statement

The actin cytoskeleton is involved in synaptic transmission and morphogenesis. However, little is known about how learning may regulate actin cytoskeleton and the putative mechanistic participation of actin cytoskeleton in memory formation. Here we show that Arp2/3 (required for actin filament branching and neuronal morphogenesis) and VASP profilin-binding domain (needed for stabilization of actin cytoskeleton and neuronal morphology) are essential for the formation of long- but not short-term fear memory in lateral amygdala. The current results provide critical evidence of how memory can be formed through controlling actin cytoskeleton structure and dynamics. Moreover, Arp2/3 and VASP profilin-binding domain may serve as targets for pharmacological treatment of fear and anxiety disorders.

## Introduction

The actin cytoskeleton is responsive to synaptic signaling, such as that triggered during learning, and consequently may mediate cellular events that underlie changes in synaptic efficacy, believed to be essential for memory formation, such as synaptic transmission and morphology (e.g., Lamprecht and LeDoux, 2004; Cingolani and Goda, 2008; Spence and Soderling, 2015; Hlushchenko et al., 2016; Lei et al., 2016). However, the roles and regulation of actin cytoskeleton in memory formation remain to be clarified.

In the present study, we were interested to elucidate whether the major actin cytoskeleton regulators Arp2/3, VASP, and formins are needed for memory formation. The actin-related protein 2/3 (Arp2/3) complex is an actin cytoskeleton nucleator that forms a new actin filament that branches off the side of a preexisting filament (Pollard, 2007; Chesarone and Goode, 2009). In neurons, Arp2/3 is concentrated in spines and is required for spine head growth and activity-dependent spine enlargement (Kim et al., 2006, 2013; Rácz and Weinberg, 2008; Wegner et al., 2008; Hotulainen et al., 2009). Formins promote elongation of linear F-actin by binding to profilin–G-actin (Pollard, 2007) and the formation of spines (Hotulainen et al., 2009). VASP recruits ATP-G-actin–bound profilin via a proline-rich region (Ferron et al., 2007). In neurons, VASP–profilin complex inhibits actin dynamics and stabilizes dendritic spine morphology (Ackermann and Matus, 2003). Thus, evidence indicates that whereas Arp2/3 and formins function facilitate spine morphogenesis, VASP and profilin, in turn, stabilize their structure (see also Bosch et al, 2014). We have shown that fear conditioning in rats leads to the translocation of profilin into dendritic spines in the lateral amygdala (LA) (Lamprecht et al., 2006).

A main unresolved question to be addressed pertains to the role of regulatory proteins that control actin cytoskeleton dynamics and neuronal morphogenesis in memory formation. Therefore, the main aim of this study was to assess whether Arp2/3, VASP, and formins play a mandatory role in the formation of memory. Toward this end, we used the fear conditioning paradigm in which an association is formed between a neutral tone conditioned stimulus (CS) and an aversive mild footshock unconditioned stimulus (US) (Fanselow and LeDoux, 1999; LeDoux, 2000; Davis and Whalen, 2001; Sah et al, 2003; Maren, 2005). The putative site of fear conditioning memory, the LA, has been identified (Fanselow and LeDoux, 1999; Schafe et al., 2001; Rodrigues et al., 2004; Maren, 2005; Johansen et al., 2011).

Our results demonstrate that Arp2/3 and VASP, but not formins, play central roles in fear memory formation in LA. The current results and supporting evidence from other studies provide the background and rationale for the proposal of a new model of how memory can be formed through controlling actin cytoskeleton structure and dynamics.

## Materials and methods

### Animals

Male Sprague-Dawley rats (250–300 g) age ∼8 weeks were used (Harlan Laboratories). Rats were housed separately at 22 ± 2°C in a 12-h light/dark cycle, with free access to food and water. The experiments were conducted during the light phase. Behavioral experiments were approved by the University of Haifa Institutional Committee for animal experiments in accordance with National Institutes of Health guidelines.

### Fear conditioning

Fear conditioning took place in a Plexiglas rodent conditioning chamber with a metal grid floor (Coulbourn Instruments, Whitehall, PA). Rats were habituated to the training chamber for 20 min 1 day before fear conditioning. On the next day, rats were allowed to acclimate to the conditioning chambers for 5 min followed by five pairings of a tone (CS; 40 s, 5 kHz, 78 dB) that was coterminated with a foot shock (US; 0.5 s, 1.3 mA). The intertrial interval (ITI) was 170–180 s. Rat groups were tested 2 h after training for short-term memory (STM) or 24 h after training for long-term memory (LTM) in a chamber with different floor, light, and odor to diminish the effect of context. Rats were given a 5 min acclimation period before the memory test and then were presented with five tones (40 s, 5 kHz, 78 dB) with ITI of 170–180 s. Behavior was recorded, and the video images were transferred to a computer equipped with FreezeFrame analysis program. The percentage of changed pixels between two adjacent 1 s images was used as a measure of activity.

### Surgical procedures

Rats were anesthetized with ketamine 0.1 ml/100 g and xylazine 0.06 ml/100 g and restrained in a stereotaxic apparatus (Kopf Instruments, Tujunga, CA). Guide stainless-steel cannulas (23 gauge) were implanted bilaterally 1.5 mm above the LA [LA coordinates are in reference to bregma: anteroposterior (AP), −3.0; lateral (L) ±5.2; and dorsoventral (DV), −8.0]. Rats were given antibiotics (penicillin and streptomycin; Norbrook Laboratories, Corby, UK) and Calmagine (Vetoquinol) for analgesia on surgery day. Rats were given 7 d for recovery before behavioral training.

### Microinjection

The stylus was removed from the guide cannula, and a 28 gauge injection cannula, extending 1.5 mm from the tip of the guide cannula aimed to the LA, was carefully placed. The injection cannula was connected via PE20 tubing and back-filled with saline with a small air bubble separating the saline from the drug solution, to a 10-μl Hamilton microsyringe, driven by a microinjection pump (PHD 2000, Harvard Apparatus, Cambridge, MA). Solution was injected at a rate of 0.5 μl/min. Total volume injected was 0.5 μl per LA. CK-666 (100 µm; Tocris Bioscience, Bristol, UK), control compound CK-689 (100 µm; Merck Millipore, Billerica, MA) or SMIFH2 (100 µm; Tocris) were dissolved in vehicle (1:1 saline and DMSO). YGRKKRRQRRRGGPPPPPGPPPPPGPPPPP-Lys(biotyn)-NH2-OH [TAT-G(GP_5_)_3_] or control peptide [YGRKKRRQRRRGGAAAAAGAAAAAGAAAAA-Lys(biotyn)-NH2-OH; TAT-G(GA_5_)_3_] (GL Biochem, Shanghai, China) were first dissolved in DMSO and then diluted with saline, to 30 µg/µl. After injection, the injection cannula was left for an additional 1 min before withdrawal to minimize dragging of injected liquid along the injection track.

### Peptide localization in brain

Rats were microinjected bilaterally with TAT-G(GP_5_)_3_ or TAT-G(GA_5_)_3_ (0.5 µl of 30 µg/µl peptide at 0.5 µl/min) and were anesthetized 30 min later using ketamine 0.1 ml/100 g and xylazine 0.06 ml/100 g. Animals were perfused intracardially with ∼250 ml PBS followed with ∼250 ml paraformaldehyde (PFA) solution (4% w/v PFA in PBS containing 5% w/v sucrose and 3.3 × 10^–4^% of 1 m, NaOH). After perfusion brains were removed and placed in 25% PFA solution in PBS and 24% w/v sucrose for 3 d at 4°C until brains sank to the bottom of the tube. Brains were frozen and sliced at a thickness of 45 μm. Slices were incubated in cold PBS for 1 h at room temperature. Slices were subjected to Alexa Fluor 568–streptavidin diluted 1:2000 in PBS. The slices were left at room temperature for 1 h to incubate. After incubation, slices were washed twice with PBS, mounted on slides, and covered with 10 µl anti-fade solution and coverslip.

### Immunohistochemistry

Animals were anesthetized by injection of ketamine and xylazine and transcardially perfused with ∼250 ml of cold 0.01 m PBS solution, followed by 250 ml of 4% PFA in 0.01 m PBS. Brains were then excised and postfixed in fixative solution containing 30% sucrose dissolved in 1% PFA in 0.01 m PBS for 2 d at 4°C. After postfixation, brains were frozen at −80°C until sectioning. Slices were prepared (50 μm thickness) using cryostats (LEICA CM 1900) and were kept floating in PBS. Sections were first kept in 0.2% Triton-X 100 in 0.01 m PBS for 30 min followed by 2 h of blocking solution containing 5% bovine serum albumin and 5% normal goat serum dissolved in 0.2% Triton X-100 solution made in 0.01 m PBS. Sections were then incubated overnight at 4°C with primary antibodies for anti-Arp3 (1:200, A5979; Sigma-Aldrich, St. Louis, MO), anti-FMNL1 (1:200, ab189940; Abcam, Cambridge, UK), or anti-profilin (1:60, APUF01; Cytoskeleton, Denver, CO) made in the same blocking solution. For colocalization of profilin and Arp3, the slices were incubated with a mixture of the anti-profilin and anti-Arp3 antibodies (keeping the same dilutions as above). After five washes in 0.01 m PBS, the slices were subjected to Alexa Fluor 488 anti-mouse secondary antibody (1:1000, A21202; Invitrogen, San Diego, CA, for Arp3), Alexa Fluor 488 anti-rabbit secondary antibody (1:500, A11008; Invitrogen, for FMNL1), or Rhodamine Red anti-rabbit secondary antibody (1:500, R6394; Invitrogen, for profilin). For colocalization of the GluA1 and Arp3 staining, sections were blocked for 1 h in blocking solution of 0.01 m PBS containing 3% bovine serum albumin and incubated overnight at 4°C with the anti-GluA1 primary antibodies that recognize the extracellular portion of the receptors (1:200, AGC-004; Alomone Labs, Jerusalem, Israel). After washing twice with 0.01 m PBS, the slices were subjected to anti-Arp3 as above followed by Alexa Fluor 488 anti-mouse secondary antibody (1:1000 for Arp3; Invitrogen) and Rhodamine Red anti-rabbit secondary antibody (1:500 for GluA1; Invitrogen) in 0.01 m PBS for 2 h at room temperature. The slices were then washed five times with 0.01 m PBS and mounted on Superfrost coated slides with Slow Fade antifade medium (Invitrogen). Slides were kept in the dark before image acquisition and analysis. Images were taken with a Nikon confocal microscope.

### Pulldown and Western blot assays

Rats were decapitated, and their brains were rapidly removed and frozen on dry ice. The lateral amygdala nucleus was punched from frozen brain with a blunted 0.5-mm diameter sample corer and stored at –80°C. The tissue was then homogenized with 300 µl homogenization buffer (150 mm sodium chloride, 1% Triton X-100, 50 mm Tris HCl, pH 8) using a glass-Teflon homogenizer and centrifuged for 5 min at 12,000 RPM at 4^o^C. Thirty µl of the supernatant and 10 µl (60 μg/μl) of peptides TAT-G(GP_5_)_3_ or TAT-G(GA_5_)_3_ were added to 20 µl streptavidin agarose beads and incubated overnight at 4°C with gentle rocking. Beads were collected by centrifugation and washed three times with 1 ml of 0.01 m PBS. After the final wash, 40 μl of sample buffer was added directly to the beads, boiled for 5 min at 80°C, and centrifuged (12,000 RPM) for 5 min at 20°C. The supernatant above the pelleted beads was loaded directly onto the gel and analyzed by Western blot. Blots were blocked with blocking buffer [5% nonfat dry milk in wash buffer (10 mm Tris, pH 7.5, 100 mm NaCl, 0.1% Tween 20)] for 2 h at room temperature. Blots were then subjected to profilin antibody (1:1000; Cytoskeleton) in blocking buffer at 4^o^C for 12 h. Blots were washed with wash buffer for 30 min. The blots were then subjected to horseradish peroxidase–conjugated anti-rabbit IgG (1:1000; Cell Signaling Technology, Danvers, MA) in blocking buffer for 1 h at room temperature. The blots were then washed three times with wash buffer for 30 min and exposed to enhanced chemiluminescence (EZ ECL) kit. Band densities were analyzed using ImageJ. The average relative density was calculated to be able to add different experiments: the density of the band from each condition in a certain experiment was divided by the density of the TAT-G(GP_5_)_3_ band from this particular experiment (experiments contained duplicates of pulldowns in most conditions).

### Histology

After behavior tests were completed, rats were decapitated and the brains were quickly removed, placed on dry ice, and stored at −80°C until use. Brains were sliced (55 μm) and stained with cresyl violet. Cannula placements were verified. Only rats with cannula tips at or within the boundaries of the LA/basolateral amygdala were included in the data analysis.

### Statistics

Data were analyzed with repeated-measures ANOVA for behavior or Kruskal–Wallis followed by Mann–Whitney *U* test analysis for biochemistry with an α level of 0.05 using IBM SPSS 21 (Table 1). Graphs show means ± SEM.

## Results

### Arp2/3 in lateral amygdala is essential for the formation of long-term fear memory

**Figure 1.**
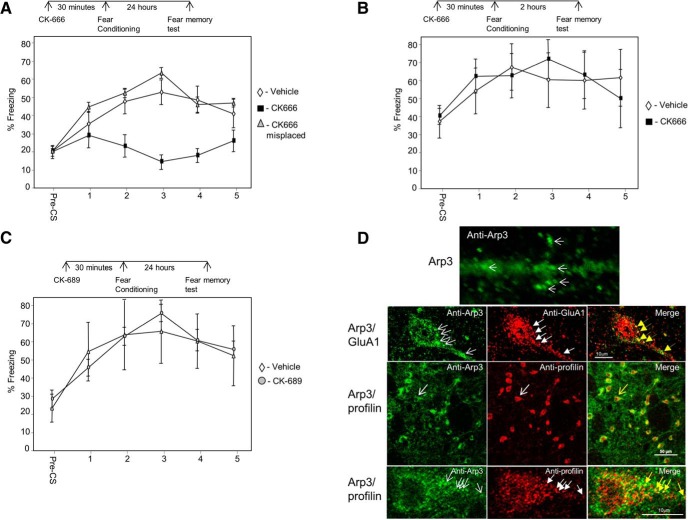
Arp2/3 in lateral amygdala is needed for long-term, but not short-term, fear memory formation. ***A***, We explored the possibility that Arp2/3 activity in LA is needed for long-term fear memory formation. Toward that end, we microinjected an Arp2/3-specific inhibitor, CK-666, into the LA 30 min before fear conditioning training and tested for fear memory 24 h after training. Animals injected with CK-666 before fear conditioning were significantly different from rats injected with vehicle or CK-666 to areas surrounding the amygdala (*F*_(2,25)_ = 5.774, *p* < 0.01). ***B***, To explore whether Arp2/3 is needed for the formation of short-term fear memory formation in LA, we microinjected CK-666 into the LA 30 min before fear conditioning training and tested for fear memory 2 h after training. Animals microinjected with CK-666 into LA were not significantly different from animals injected with vehicle (*F*_(1,13)_ = 0.035, *p* > 0.8). ***C***, To test whether the effects of CK-666 are caused by the injection of a compound per se, we microinjected an inactive control compound CK-689 into LA and compared its effects to injection of vehicle. Long-term fear memory in animals microinjected with CK-689 was not significantly different from animals microinjected with vehicle (*F*_(1,8)_ = 0.002, *p* > 0.9). ***D***, Upper panel, high magnification of Arp3 labeling in dendrite in LA. Arp3 is found in puncta (arrows). Second panel Arp3 (green) and GluA1 (red) are colocalized (yellow in merged). Arrows point at examples of protein puncta that are colocalized. Third panel, Arp3 (green) and profilin (red) are colocalized (yellow in merged) in LA. Arrows indicate the neuron enlarged in lowest panel. Lower panel, enlarged neuron showing colocalization of profilin and Arp3. Arrows, example for colocalized proteins.

The major actin nucleator Arp2/3 complex is needed for actin filament branching (Pollard, 2007). To explore whether Arp2/3 is needed for the formation of LTM in LA, we microinjected an Arp2/3-specific inhibitor, CK-666 (Nolen et al., 2009), and tested fear memory formation 24 h after training. Animals injected with CK-666 (*n* = 9) before fear conditioning were significantly different in fear conditioning LTM from rats injected with vehicle (*n* = 12) or injected with CK-666 to areas surrounding the amygdala (*n* = 7) (*F*_(2,25)_ = 5.774, *p* < 0.01) (Fig. 1*A*). The treatment × tone trial interaction was not significant (*F*_(5.934,74.174)_ = 1.877, *p* > 0.09). Cannula placements for all experiments are shown in Figure 4. Thus, the results show that Arp2/3 in LA is essential for long-term fear memory formation.

### Arp2/3 in lateral amygdala is not required for the formation of short-term fear memory

To explore whether Arp2/3 is needed for the formation of STM in LA, CK-666 was microinjected into the rat LA 30 min before fear conditioning training, and fear memory was tested 2 h after training. Rats microinjected with CK-666 into LA (*n* = 8) were not significantly different from animals injected with vehicle (*n* = 7) (*F*_(1,13)_ = 0.035, *p* > 0.8) (Fig. 1*B*). The treatment × tone trial interaction was not significant (*F*_(1.899,24.690)_ = 0.78, *p* > 0.4). These results show that Arp2/3 in LA is not needed for short-term fear memory formation.

### Inactive control compound CK-689 injected into the LA has no effect on long-term fear memory formation

To test whether the effects of CK-666 are caused by the injection of a compound per se, we microinjected an inactive control compound CK-689 (Nolen et al., 2009) into LA and compared its effects to injection of vehicle. CK-689 lacks both the 2-methyl on the indole ring and most of the thiophene ring, eliminating favorable interactions with Arp2 and Arp3. Long-term fear memory in rats microinjected with CK-689 (*n* = 5) was not significantly different from animals microinjected with vehicle (*n* = 5) (*F*_(1,8)_ = 0.002, *p* > 0.9) (Fig. 1*C*). The treatment × tone trial interaction was not significant (*F*_(1.785,14.282)_ = 0.631, *p* > 0.5). This result shows that injection of the inactive control compound CK-689, devoid of inhibitory action, has no effect on long-term fear memory formation. Cumulatively, the aforementioned observations show that the Arp2/3 is essential for consolidation, but not for the acquisition, of fear memory in LA.

### Arp2/3 is colocalized in lateral amygdala with GluA1 and profilin

To gain additional insights into the mode of function of Arp2/3 in LA, we performed immunohistochemistry experiments to detect Arp3 localization in cells in LA. Arp3 protein is found to be enriched in puncta in dendrites, spines, and soma of neurons (Fig. 1*D*). To characterize these clusters, we performed colocalization studies of Arp2/3 with GluA1 subunit of AMPA receptor. GluA1 containing synapses in LA are the main synapses receiving excitatory inputs from the auditory thalamus and auditory cortex (Farb and LeDoux, 1997, 1999), mostly on dendritic spines (Farb and LeDoux, 1997). These pathways are essential for fear conditioning (LeDoux, 2000). In addition, GluA1 in LA is needed for fear conditioning memory formation (Rumpel et al., 2005). Figure 1*D* shows that Arp3 is colocalized with GluA1, suggesting that Arp2/3 functions affect GluA1-containing synapses. We further studied whether Arp2/3 is localized with profilin. Observations suggest that spines that are enlarged by Arp2/3 are subsequently stabilized by profilin (Ackermann and Matus, 2003; Bosch et al., 2014; Michaelsen-Preusse et al., 2016). It was also observed that profilin is translocated into spines of animals that are trained for fear conditioning (Lamprecht et al., 2006). Moreover, it was shown that these profilin-containing spines are larger than those that lack profilin. Therefore, a tenable hypothesis is that spines that are enlarged after fear conditioning by Arp2/3 are stabilized subsequently by profilin (see Discussion). A prerequisite for such a possibility is that Arp2/3 is colocalized with profilin in LA neurons. Animals were trained for fear conditioning, and the localization of Arp3 and profilin in LA was examined 30 min after training. Figure 1*D* shows that Arp3 and profilin colocalize in puncta in neurons, suggesting that Arp2/3 and profilin may act to enlarge and consequently stabilize the synapses in LA, respectively. We next examined whether VASP–profilin interaction, needed for translocation of profilin into spines and to their stabilization (Ackermann and Matus, 2003), is necessary for fear memory formation.

### VASP is needed in lateral amygdala for long-term fear memory formation

**Figure 2.**
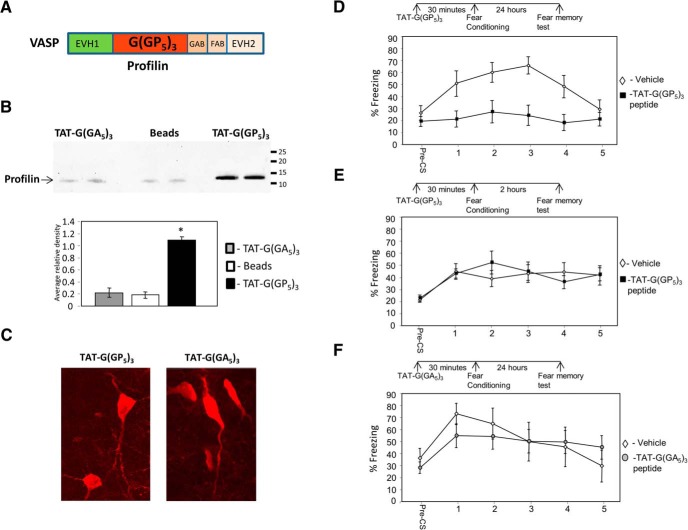
VASP–profilin binding domain is needed in lateral amygdala for long-term, but not short-term, fear memory formation. ***A***, Schematic drawing of VASP domains including the profilin poly-proline binding domain. ***B***, TAT-G(GP_5_)_3_ binds profilin in amygdala homogenate. In contrast, a control peptide containing poly-alanine TAT-G(GA_5_)_3_ or beads alone do not. TAT-G(GP_5_)_3_ pulled down significantly more profilin (*p* < 0.002) than TAT-G(GA_5_)_3_ (*p* < 0.0002) or beads (*p* < 0.002). ***C***, Microinjection of TAT-G(GP_5_)_3_ or TAT-G(GA_5_)_3_ peptides into LA led to their internalization into cells when tested 30 min later. ***D***, Animals were microinjected with TAT-G(GP_5_)_3_ into the LA 30 min before fear conditioning training and tested for fear memory 24 h after training for fear LTM. Fear conditioning memory in TAT-G(GP_5_)_3_-microinjected rats was significantly different from that in animals injected with vehicle (*F*_(1,21)_ = 14.609, *p* < 0.002). ***E***, To study whether the VASP is needed for STM formation, we microinjected TAT-G(GP_5_)_3_ into the LA 30 min before fear conditioning and tested for fear memory 2 h after training. Animals microinjected with TAT-G(GP_5_)_3_ into LA were not significantly different from animals injected with vehicle (*F*_(1,13)_ = 0.087, *p* > 0.7). ***F***, To test whether the effects of TAT-G(GP_5_)_3_ are caused by the injection of a peptide per se, we microinjected a TAT-control peptide, TAT-G(GA_5_)_3_, into LA and compared its effects to injection of vehicle. There was no significant difference between animals injected with TAT-G(GA_5_)_3_ peptide and vehicle when tested for long-term fear memory (*F*_(1,18)_ = 0.031, *p* > 0.8).

It was shown that profilin is translocated into dendritic spines after NMDA receptors stimulation and by stimuli creating long-term potentiation (LTP) or long-term depression (LTD; Ackermann and Matus, 2003), leading to suppression of actin dynamics and stabilization of spine morphology. VASP binding to profilin, through its polyproline motif (Fig. 2*A*), is required for glutamate-induced translocation of profilin into dendritic spines and for consolidation and stabilization of spine morphology (Ackermann and Matus, 2003). It has been shown that profilin translocates into dendritic spines in LA after fear conditioning (Lamprecht et al., 2006). We were therefore interested to explore the roles of VASP in LA in fear conditioning by introducing a polyproline peptide that competes with VASP binding to profilin (Reinhard et al., 1995). This peptide binds profilin and inhibits actin-dependent motility (Kang et al., 1997) and glutamate-induced profilin translocation into dendritic spines (Ackermann and Matus, 2003). The peptide [G(GP_5_)_3_] is conjugated to TAT [TAT-G(GP_5_)_3_] to facilitate its delivery into cells (Schwarze et al., 1999). We first tested whether TAT-G(GP_5_)_3_ can bind profilin. Figure 2*B* shows that TAT-G(GP_5_)_3_ binds profilin in amygdala homogenate. In contrast, a control peptide containing poly-alanine TAT-G(GA_5_)_3_ or beads alone are ineffective in this respect (Fig. 2*B*). TAT-G(GP_5_)_3_ pulled down significantly more profilin (*p* < 0.002) than TAT-G(GA_5_)_3_ (*p* < 0.0002) or beads (*p* < 0.002). Microinjection of TAT-G(GP_5_)_3_ or TAT-G(GA_5_)_3_ peptides into LA led to their internalization into cells when tested 30 min later (Fig. 2*C*). Next, we explored the possibility that VASP–profilin binding site in LA is needed for long-term fear memory formation. Toward this end, we microinjected TAT-G(GP_5_)_3_ into the LA 30 min before fear conditioning training and tested for fear LTM 24 h after training. Fear conditioning memory in TAT-G(GP_5_)_3_-microinjected rats (*n* = 12) was significantly impaired compared to animals injected with vehicle (*n* = 11) (*F*_(1,21)_ = 14.609, *p* < 0.002) (Fig. 2*D*). The treatment × tone trial interaction was significant (*F*_(4,84)_ = 3.001, *p* < 0.024). This result shows that VASP function, through its profilin binding site, in LA is needed for long-term fear memory formation.

### VASP is not required for the formation of short-term fear memory in lateral amygdala

To study whether VASP is needed for STM formation, we microinjected TAT-G(GP_5_)_3_ into the LA 30 min before fear conditioning and tested for fear memory 2 h after training. Animals microinjected with TAT-G(GP_5_)_3_ into LA (*n* = 7) were not significantly different from animals injected with vehicle (*n* = 8) (*F*_(1,13)_ = 0.087, *p* > 0.7) (Fig. 2*E*). The treatment × tone trial interaction was not significant (*F*_(4,52)_ = 1.013, *p* > 0.4). This result shows that VASP function, through its profilin binding site, in LA is not needed for short-term fear memory formation.

### Control peptide injected into the LA has no effect on long-term fear memory

To test whether the effects of TAT-G(GP_5_)_3_ are caused by the injection of a peptide per se, we microinjected a TAT-control peptide, TAT-G(GA_5_)_3,_ into LA and compared its effects to injection of vehicle. There was no significant difference between animals injected with TAT-G(GA_5_)_3_ peptide (*n* = 11) and vehicle (*n* = 9) when tested for long-term fear memory (*F*_(1,18)_ = 0.031, *p* > 0.8) (Fig. 2*F*). The treatment × tone trial interaction was not significant (*F*_(4,72)_ = 1.620, *p* > 0.17). These results show that injection of a peptide per se has no effect on LTM fear memory formation. Taken together, the aforementioned observations show that VASP, through its profilin binding site, is needed for the consolidation, but not acquisition, of fear memory formation in LA.

### Formins are not needed for LTM in lateral amygdala

**Figure 3.**
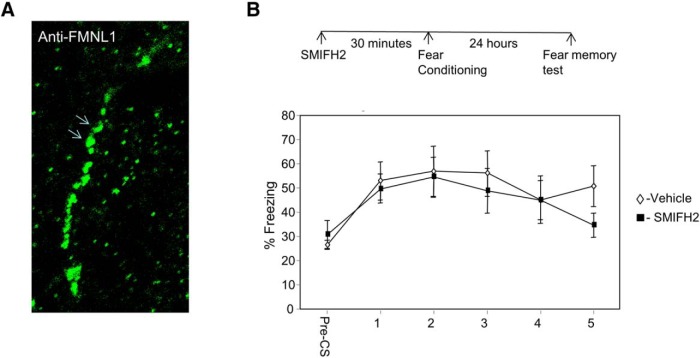
Formin is not needed for long-term memory in lateral amygdala. ***A***, Formins are located in neurons in amygdala. Arrows show the localization of FMNL1 in neuronal dendrite in LA. ***B***, Microinjection of formins inhibitor SMIFH2 into the LA 30 min before fear conditioning had no effect on long-term fear memory formation compared to controls (*F*_(1,23)_ = 0.317, *p* > 0.5).

Linear actin cytoskeleton polymerization can be facilitated by formins (Pollard, 2007; Ferron et al., 2007). Formins recruit profilin-bound G-actin to regulate F-actin. The aforementioned results have shown that profilin binding site on VASP is essential for the formation of fear memory in LA. We show that formins are located in neurons in amygdala. Figure 3*A* shows the localization of the formin FMNL1 in neuronal dendrite in LA. We were therefore interested to explore whether formins in LA are needed for fear memory formation. Toward that end, we microinjected a formin-specific inhibitor, SMIFH2 (Rizvi et al., 2009), into the LA and studied its effects on long-term fear memory. Microinjection of SMIFH2 into LA (*n* = 14) 30 min before fear conditioning had no effect on long-term fear memory formation compared to controls (*n* = 11) (*F*_(1,23)_ = 0.317, *p* > 0.5). The treatment × tone trial interaction was not significant (*F*_(3.187,73.307)_ = 0.861, *p* > 0.4). This result shows that formins are not needed for long-term memory formation in LA.

**Figure 4.**
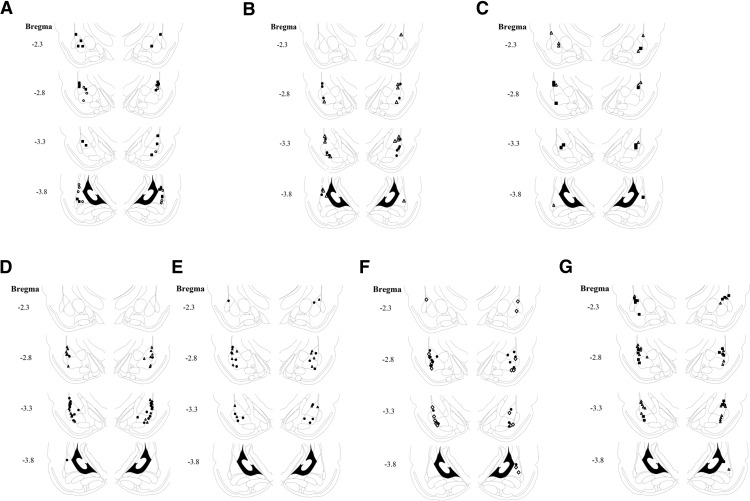
Cannula placements. ***A***, Cannula tip placements from rats injected with CK-666 tested for LTM. Filled square, vehicle; open circle, CK-666. ***B***, Cannula tip placements from rats injected with CK-666 tested for STM. Filled circle, vehicle; open triangle, CK-666. ***C***, Cannula tip placements from rats injected with CK-689 tested for LTM. Filled square, vehicle; open triangle, CK-689. ***D***, Cannula tip placements from rats injected with TAT-G(GP_5_)_3_ tested for LTM. Filled circle, vehicle; open triangle, TAT-G(GP_5_)_3_. ***E***, Cannula tip placements from rats injected with TAT-G(GP_5_)_3_ tested for STM. Filled circle, vehicle; open triangle, TAT-G(GP_5_)_3_. ***F***, Cannula tip placements from rats injected with TAT-G(GA_5_)_3_ tested for LTM. Filled circle, vehicle; open diamond, TAT-G(GA_5_)_3_. ***G***, Cannula tip placements from rats injected with SMIFH2 tested for LTM. Filled square, vehicle; open triangle, SMIFH2.

## Discussion

In this study, we aimed to unveil possible mechanisms whereby actin cytoskeleton is regulated in LA to subserve long-term memory formation. We study whether the nucleator actin-binding protein Arp2/3, which leads to branched actin filaments, and formins and VASP, which regulate linear actin filaments, are needed in LA for memory formation. Our results show that Arp2/3 and VASP, through the profilin-binding site, but not formins, are essential for long-term, but not short-term, fear memory formation in LA.

Arp2/3 is the major actin nucleator in neurons that forms a new actin filament that branches off the side of a preexisting filament (Pollard, 2007). We observed that Arp2/3 is essential for long-term but not short-term fear memory formation. These results suggest that Arp2/3 activity in LA does not affect synaptic transmission and brain faculties needed for CS-US association in LA, as 0.002 Arp2/3 inhibition had no effect on short-term memory formation. Alternatively, it is plausible that Arp2/3 activity is required for long-lasting neuronal alterations needed for memory consolidation in LA, rather than synaptic transmission involved in memory acquisition. Ample evidence suggests that alterations of synapse and spine morphology underlie enduring changes in synaptic efficacy that subserve long-term memory (Lamprecht and LeDoux, 2004; Bailey et al., 2015). It was shown that Arp2/3 complex is concentrated in spines (Rácz and Weinberg, 2008) and that it is required for spine head growth (Wegner et al., 2008; Hotulainen et al., 2009) and for activity-dependent spine enlargement (Kim et al., 2006, 2013). Thus, these observations, together with previous results showing that fear conditioning leads to increase in spines size in LA (Lamprecht et al., 2006; Ostroff et al., 2010), suggest that Arp2/3 is required for changes in spine morphology during fear memory formation. Interestingly, Arp2/3 is shown to be involved in memory formation in *C*aenorhabditis *elegans* (Hadziselimovic et al., 2014). In that study, the authors show that musashi (msi-1) is necessary for time-dependent memory loss. They show that MSI-1 binds to mRNAs of three subunits of the Arp2/3 complex and downregulates ARX-1, ARX-2, and ARX-3 translation upon associative learning. Increase in Arp2/3 complex activity in the AVA interneuron inhibits memory loss. Moreover, inhibition of the Arp2/3 complex activity by CK-666 suppresses the enhanced memory phenotype of msi-1(lf).

A key question that arises is how neuronal structural changes that are induced by learning are stabilized for long periods of time to consolidate memory formation. One potential protein that can mediate stabilization of neuronal morphology is profilin (Ackermann and Matus, 2003; Michaelsen et al., 2010; Michaelsen-Preusse et al., 2016). It was shown that profilin is translocated into dendritic spines after NMDA receptor stimulation and stimuli leading to LTP or LTD (Ackermann and Matus, 2003). Such translocation of profilin starts minutes after stimulation and lasts for many hours. The movement of profilin into dendritic spines leads to suppression of actin dynamics and stabilization of spine morphology. Profilin–G-actin complex binds to VASP through its poly‐proline segment [G(GP_5_)_3_] (Reinhard et al., 1995; Ferron et al., 2007) and this VASP binding to profilin is required for glutamate-induced translocation of profilin into dendritic spines and consolidation and stabilization of spine morphology (Ackermann and Matus, 2003). It has been shown that profilin translocates into dendritic spines in LA after fear conditioning (Lamprecht et al., 2006). These spines that contained profilin in LA are larger than spines that did not contain profilin. We were therefore interested to explore whether introducing a peptide, G(GP_5_)_3_, in LA would impair fear memory formation. This peptide competes with profilin binding to VASP and inhibits translocation of profilin into spines (Reinhard et al., 1995; Ackermann and Matus, 2003). We show that microinjection of G(GP_5_)_3,_ but not the control peptide G(GA_5_)_3_, impaired long-term but not short-term fear memory formation in LA. These results show that VASP–profilin binding in LA cells is essential for fear memory formation and indicate a functional significance of VASP–profilin interaction in LA. Taken together, the aforementioned results suggest that translocation of profilin into spines mediated by VASP after fear conditioning leads to suppression of actin dynamics in spines and long-term stabilization of spine morphology. Such stabilization of synaptic and spine morphology is suggested to mediate the formation of long-term memory (Lamprecht and LeDoux, 2004; Bailey et al., 2015).

Formins bind profilin to elongate actin filaments. Actin–profilin complex binds to multiple sites on the FH1 domain on formin and is transferred rapidly to the growing barbed end associated with the FH2 domain on formin (Pollard, 2007; Goode and Eck, 2007). Our results show that formins are found in LA. However, although VASP and formins control the dynamics of actin filaments via profilin, we show that in LA VASP, but not formins, is required for fear memory formation. Interestingly, formin was shown to be involved in age-dependent memory formation (Peleg et al., 2010). Formin 2 protein levels transiently increased in response to contextual fear conditioning in 3-month-old, but not 16-month-old, mice. Three- and 8-month-old formin 2^–/–^ mice and wild-type littermates were subjected to contextual fear conditioning. Although the 3-month groups showed similar freezing behavior, 8-month-old formin 2^–/–^ mice displayed impaired associative learning compared with age-matched wild-type littermates. It would be interesting to examine whether formins have an effect in amygdala in older rats.

Previous observations show that profilin is found in larger spines in LA after fear conditioning (Lamprecht et al., 2006) and that profilin stabilizes spine morphology (Ackermann and Matus, 2003). Thus, these spines should be enlarged before profilin translocates into them, leading to their stabilization. Arp2/3 protein is the central protein mediating spine enlargement through actin branching (Kim et al., 2006, 2013; Rácz and Weinberg, 2008; Wegner et al., 2008; Hotulainen et al., 2009). We therefore suggest that Arp2/3-mediated actin filament branching precedes stabilization induced by profilin. In this model, fear conditioning leads to actin polymerization and actin filament branching mediated by Arp2/3. These alterations in actin filaments lead to enlargement in spine morphology. Spine enlargement is stabilized subsequently by the translocation of profilin into dendritic spines and suppression of actin dynamics. These changes in neuronal morphogenesis are not needed for short-term memory but specifically for the consolidation of long-term memories. Consistent with our model is a study showing that Arp2/3 amounts in activated spine are increased rapidly (1–7 min) after activation in correlation with spine enlargement (Bosch et al., 2014). This initial phase is followed by translocation of profilin into spines and spine structure stabilization. Moreover, the model is further supported by the observation that profilin is colocalized with Arp2/3 in LA of fear-conditioned rats. However, additional morphological evidence will be needed to support this model.

In the present study, we show for the first time that Arp2/3 and VASP, but not formins, are needed in LA for long-term, but not short-term, fear memory formation. The results show that the actin cytoskeleton needed for fear memory formation (Mantzur et al., 2009; Rehberg et al., 2010; Gavin et al., 2011) is tightly regulated in LA by specific regulatory proteins controlling its branching and stabilization. Our results suggest a mechanism whereby actin cytoskeleton remodeling leads to changes in neuronal morphogenesis needed for memory consolidation.

**Table 1.** Summary of results and statistical analysis

Figure	*p*-value	Type of test
1*A*	0.009	Repeated-measures ANOVA, between-subjects effects
1*A*	0.097	Repeated-measures ANOVA, treatment × tone trial interaction
1*B*	0.855	Repeated-measures ANOVA, between-subjects effects
1*B*	0.463	Repeated-measures ANOVA, treatment × tone trial interaction
1*C*	0.964	Repeated-measures ANOVA, between-subjects effects
1*C*	0.529	Repeated-measures ANOVA, treatment × tone trial interaction
2*B*	0.001	Kruskal–Wallis
2*B*	0.000155	Mann–Whitney *U*, TAT-G(GP_5_)_3_ vs. TAT-G(GA_5_)_3_
2*B*	0.001	Mann–Whitney *U*, TAT-G(GP_5_)_3_ vs. beads
2*B*	0.573	Mann–Whitney *U*, TAT-G(GA_5_)_3_ vs. beads
2*D*	0.001	Repeated-measures ANOVA, between-subjects effects
2*D*	0.023	Repeated-measures ANOVA, treatment × tone trial interaction
2*E*	0.773	Repeated-measures ANOVA, between-subjects effects
2*E*	0.409	Repeated-measures ANOVA, treatment × tone trial interaction
2*F*	0.862	Repeated-measures ANOVA, between-subjects effects
2*F*	0.179	Repeated-measures ANOVA, treatment × tone trial interaction
3*B*	0.579	Repeated-measures ANOVA, between-subjects effects
3*B*	0.471	Repeated-measures ANOVA, treatment × tone trial interaction

## Synthesis

Reviewing Editor: Thomas McHugh, RIKEN Brain Science Institute. Decisions are customarily a result of the Reviewing Editor and the peer reviewers coming together and discussing their recommendations until a consensus is reached. When revisions are invited, a fact-based synthesis statement explaining their decision and outlining what is needed to prepare a revision will be listed below.

Based on the authors' response and changes to the earlier reviews I can tentatively accept this manuscript without further review or major revisions. A few minor changes and addition of a missing items are reuiqred to finalize the manuscript:

1) eNeuro requires a stand-alone table reporting all statistical tests and results (please see http://eneuro.org/information-for-authors#materials or any publihsed eNeuro article for an example) Please prepare this.

2) In the abstract the authors should not use the abbreviation LA prior to defining it

3) in the introduction the authors define LA twice (line 88 and line 97 on pg.3), in the second instance please just use LA

4) Commas in the first sentence of the paragraph beginning on line 90 of pg. 3 are unnecessary, please remove.
